# Real-World Clinical Experience With Monthly Faricimab for Treatment-Naïve Diabetic Macular Edema: A Case Series From an Indian Tertiary Care Center

**DOI:** 10.7759/cureus.88475

**Published:** 2025-07-21

**Authors:** Anand K Bukke, Uma Ganganakurthi, Rajalingam Vairagyam, Keerthi R Vontela

**Affiliations:** 1 Department of Retina, Sarojini Devi Eye Hospital, Hyderabad, IND

**Keywords:** angiopoietin-2, anti-vegf, best-corrected visual acuity, central macular thickness, diabetic macular edema, faricimab, treatment-naïve

## Abstract

This case series explores the efficacy and safety of a monthly loading dose regimen of intravitreal faricimab in treatment-naïve patients with diabetic macular edema (DME) in an Indian clinical setting.

In this retrospective analysis, five adult patients with treatment-naïve DME received faricimab (four intravitreal injections of 6 mg) on Day 0, Day 30, Day 60, and Day 90. Their best-corrected visual acuity (BCVA) and central macular thickness (CMT) were evaluated throughout the study. Additional outcome measures included adverse events and patient-reported treatment satisfaction, graded from "very satisfied" to "not satisfied." All patients exhibited notable improvements in BCVA alongside significant reductions in CMT over the three-month follow-up period, achieving marked anatomical recovery. Visual gains were notably greater in patients with a shorter duration of diabetes and better baseline vision. No ocular adverse events were observed during the study. Overall, patient satisfaction scores improved from "moderate" at baseline to "very satisfied" by the end of follow-up. These findings suggest that monthly loading doses of intravitreal faricimab are favorable for treating treatment-naïve DME patients. The results reinforce faricimab’s potential to improve disease management, offering both safety and efficacy in real-world clinical settings.

## Introduction

Diabetic macular edema (DME) is a multifactorial disorder of the macula, resulting from various pathological mechanisms in the retina and its interaction with complementary pathways. DME is a leading cause of irreversible visual impairment and blindness [1]. Type 2 diabetes mellitus (T2DM) is reaching epidemic proportions globally, affecting 382 million people, with numbers expected to rise to 783.2 million by 2045 [1]. As a complication of diabetic retinopathy, DME results from fluid accumulation in the macula, leading to metamorphopsia and potentially severe loss of visual function if untreated. The United States Administrative Medical Claims Database (2000-2022) documented an initial decline in DME prevalence from 2001 to 2007, followed by a steady annual increase from 2007 (3.2%) to 2016 (5.4%), and a subsequent annual decrease through 2021 (4.9%). Similarly, the incidence rates markedly increased over the past two decades, with peaks in 2009 (8.6%) and a decline through 2022 (5%) [2]. In India, the prevalence of DME ranges between 2.4% and 8.9% [3].

The impact of DME extends beyond visual acuity loss. Deterioration in central vision adversely affects daily activities such as driving and reading, significantly impairing the quality of life of working-age individuals. While anti-vascular endothelial growth factor (VEGF) agents have revolutionized treatment for retinal vascular diseases, a substantial proportion of patients with DME do not achieve optimal outcomes. Up to 40% of eyes treated with anti-VEGF monotherapies fail to respond adequately [4]. Concerns also remain regarding the appropriate dosing, injection intervals, and long-term safety of these treatments. The necessity for multiple intravitreal injections not only prolongs treatment duration but also imposes economic and psychological burdens, alongside risks of ocular adverse effects such as a sustained increase in intraocular pressure, retinal pigment epithelium tears, and geographic atrophy [5]. DME remains a major unmet challenge in managing retinal diseases in modern times [4].

Faricimab inhibits both VEGF-A and angiopoietin-2 (Ang-2) and targets complementary pathways that regulate vascular permeability and stability. The presence of Ang-2 significantly enhances VEGF-A-induced vascular permeability, suggesting a synergistic interaction between these pathways. This highlights the pivotal role of Ang-2 in disrupting vascular integrity in diabetic retinopathy [6]. Faricimab, by simultaneously inhibiting both pathways, has shown superior efficacy in improving visual and anatomical outcomes in patients with DME versus VEGF inhibition alone. Additionally, it may extend treatment intervals and reduce the overall treatment burden [7]. The prolonged pharmacodynamic effects of faricimab further contribute to the durability of the treatment [8]. Furthermore, the treat-and-extend (T&E) regimen of faricimab is linked to fewer injections than other dosing strategies, suggesting a reduced treatment burden and more sustained therapeutic effects, particularly after an initial loading phase. Following this phase, intervals can be gradually extended, facilitating interval extension in subsequent treatments [9].

Recent real-world evidence from India has highlighted the potential benefits of a monthly loading dose regimen of faricimab. The study showed that treatment-naïve and recalcitrant patients with DME experienced significant improvements in best-corrected visual acuity (BCVA), with the naïve group exhibiting notably greater gains [10]. Each patient received three monthly loading doses followed by an as-needed (pro re nata) regimen, showing the practical and clinical relevance of this dosing strategy in routine clinical practice [10]. Limited real-world data currently exist on faricimab [10]; hence, the present case series aimed to address this gap by reporting the outcomes associated with a monthly loading dose regimen.

## Case presentation

Methods

This retrospective case series analysis was conducted at a single center, Sarojini Devi Eye Hospital, Hyderabad, a tertiary care eye hospital in India, and included five treatment-naïve adult patients diagnosed with DME with diminution of vision. Institutional Ethical Approval, dated 1 November 2024 and bearing the number ECR/300/inst/AP/2013/RR-19, was obtained from the Institutional Ethics Committee of Osmania Medical College. Patients provided informed consent for the use of their medical data in publications and presentations for scientific purposes. Individuals who had participated in any investigational ophthalmology trial involving an experimental drug or procedure within 28 days before enrollment were excluded. All patients in this study were treatment-naïve for their condition and had not received any prior treatment. A four-week dosing regimen was chosen for loading doses in line with the prescribing information for faricimab.

The treatment protocol consisted of four intravitreal faricimab injections (6 mg) given as loading doses on Day 0, Day 30, Day 60, and Day 90. The first patient’s initial visit occurred on 22 June 2024, and the follow-up period was 3 months. The primary outcome measure was the change in BCVA from baseline, measured at baseline, 1 month, 2 months, and 3 months using Snellen or Early Treatment Diabetic Retinopathy Study (ETDRS) charts. Secondary outcomes included changes in central macular thickness (CMT) over time measured with optical coherence tomography (OCT), the mean number of faricimab injections at 3 months, adverse events (AEs) throughout the study, and patient-reported treatment satisfaction categorized as very satisfied, moderately satisfied, a little satisfied, not satisfied, or uncertain.

Results

The mean (SD) age of the patients was 51.7 (7.5) years, and the mean (SD) disease duration was 1.4 (0.7) months. The mean (SD) random blood sugar level at baseline was 136.4 (19.9) mg/dL. All patients received a standardized treatment regimen of monthly intravitreal faricimab injections for three months. Further patient characteristics and baseline details are summarized in Table 1.

**Table 1:** Baseline demographics and clinical characteristics of the study participants. BCVA: Best-corrected visual acuity; BP: Blood pressure; CAD: Coronary artery disease; DME: Diabetic macular edema; ETDRS: Early Treatment Diabetic Retinopathy Study; HTN: Hypertension; mmHg: Millimeters of mercury (unit of blood pressure); mg/dL: Milligrams per deciliter (unit of blood glucose measurement); PTCA: Percutaneous transluminal coronary angioplasty; RBS: Random blood sugar; S/P: Status post.

Patient No.	Eye Examined	Age (years)	Gender	BCVA at Baseline (Snellen or ETDRS)	History of Diabetes Mellitus	Duration of DME	Comorbidities	BP (mmHg)	RBS (mg/dL)
1	Right	43	Male	3/60	1 year	1 month	None	100/60	105
2	Right	58	Female	6/60	13 years	3 months	HTN (15 years), CAD (15 years)	120/80	160
3	Right	58	Male	6/60	10 years	1 month	HTN (10 years)	110/65	135
4	Right	54	Female	1/60	25 years	2 months	CAD (S/P PTCA)	118/82	146
5	Right	44	Male	6/18	1 year	1 month	None	120/68	134

Case reports

The summary of patient outcomes is described in Table 2.

**Table 2:** Summary of patient outcomes. CMT: Central macular thickness; µm: Micrometer.

Patient	Age (years) / Gender	Baseline Vision → 3-Month Vision	Baseline CMT → 3-Month CMT (µm)	Patient Satisfaction (Start → End)
1	43 / Male	3/60 → 6/9	364 → 231	Moderate → Very satisfied
2	58 / Female	6/60 → 6/6	378 → 256	Moderate → Very satisfied
3	58 / Male	6/60 → 6/6	392 → 210	Moderate → Very satisfied
4	54 / Female	1/60 → 6/12	487 → 209	Slight → Very satisfied
5	44 / Male	6/18 → 6/6	565 → 279	Moderate → Very satisfied

Case 1 

**Figure 1:**
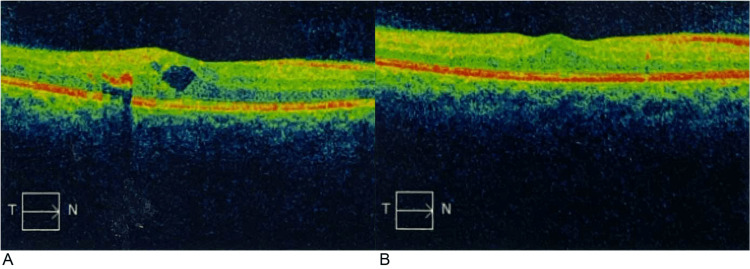
Visual representation (OCT scans: T→N) of treatment response in Patient 1. (A) Baseline; (B) Follow-up at 1 month – central macular thickness of 310 µm. T→N: Temporal to nasal orientation; OCT: Optical Coherence Tomography.

The patient presented with 1 month of diminution of vision alongside a 1-year history of DM. Diagnostic evaluation confirmed DME. Visit 1 assessment revealed counting fingers at 3 m (3/60) BCVA and 364 μm CMT. Following monthly faricimab injections, BCVA improved progressively: 6/60 at 1 month (CMT 310 μm), 6/18 at 2 months (CMT 290 μm), and 6/9 at 3 months (CMT 231 μm). Concomitant medications included oral hypoglycemics, Nevanac, and moxifloxacin. Patient satisfaction improved from moderate (score 2) at early follow-ups to very satisfied (score 1) by 3 months. The case is visually represented in Figure 1.

Case 2

**Figure 2:**
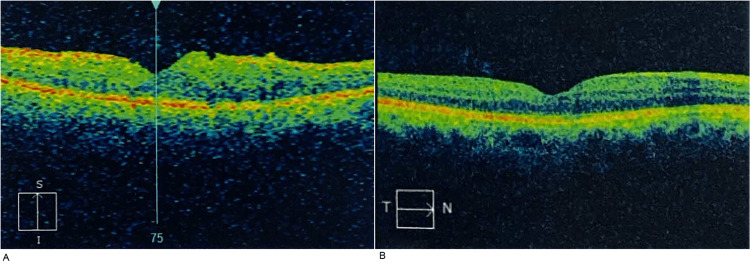
Visual representation (OCT scans: T→N) of treatment response in Patient 2. (A) Baseline: central macular thickness of 378 µm; (B) Follow-up at 2 months: thickness of 272 µm. S→I: Superior to inferior; T→N: Temporal to nasal orientation; OCT: Optical Coherence Tomography.

This patient, with a 13-year history of DM and hypertension (HTN), had experienced diminution of vision for 3 months before presentation. Initial DME evaluation at baseline revealed BCVA 6/60 and CMT 378 μm. Monthly faricimab administration resulted in improved gains: BCVA 6/36 (CMT 347 μm) at 1 month, 6/18 (CMT 272 μm) at 2 months, and 6/6 (CMT 256 μm) at 3 months. Concomitant systemic therapy included antihypertensives, aspirin, and hypoglycemics. Satisfaction scores improved from moderate (score 2 at 1- and 2-month follow-ups) to very satisfied (score 1) by the end of treatment. No AEs were reported. The case is visually represented in Figure 2.

Case 3

**Figure 3:**
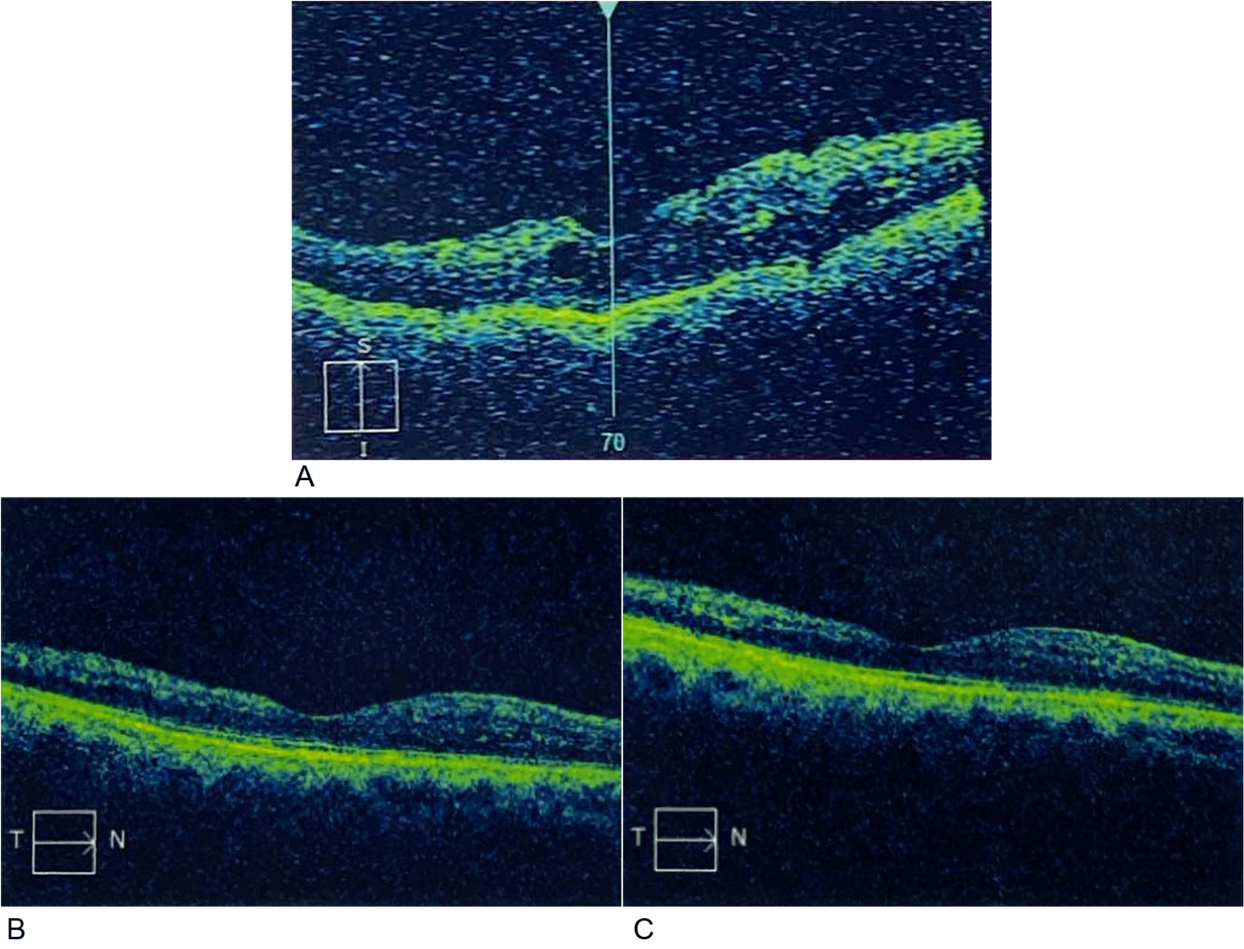
Visual representation (OCT scans: T→N) of treatment response in Patient 3. (A) Baseline: central macular thickness of 392 µm; (B) Follow-up at 1 month: thickness of 237 µm; and at 2 months: thickness of 214 µm. S→I: Superior to inferior; T→N: Temporal to nasal orientation; OCT: Optical Coherence Tomography.

This patient, with a 10-year DM/HTN history, demonstrated a rapid anatomical response to faricimab, with CMT reduction from 392 μm to 210 μm over three cycles. He presented with a 1-month history of diminished vision, leading to the diagnosis of DME. Monthly faricimab injections improved outcomes as follows: at 1 month, BCVA improved from 6/60 (baseline) to 6/18, with CMT decreasing to 237 μm. By 2 months, BCVA improved to 6/9, and CMT further reduced to 214 μm. At 3 months, BCVA reached 6/6, with CMT slightly reduced to 210 μm. The patient concomitantly continued oral hypoglycemics, antihypertensives, Nevanac, and moxifloxacin eye drops. Initially, he reported being moderately satisfied (score 2), which improved to very satisfied (score 1) after the first dose and remained consistent through all follow-ups. No AEs were reported. The case is visually represented in Figure 3.

Case 4

**Figure 4:**
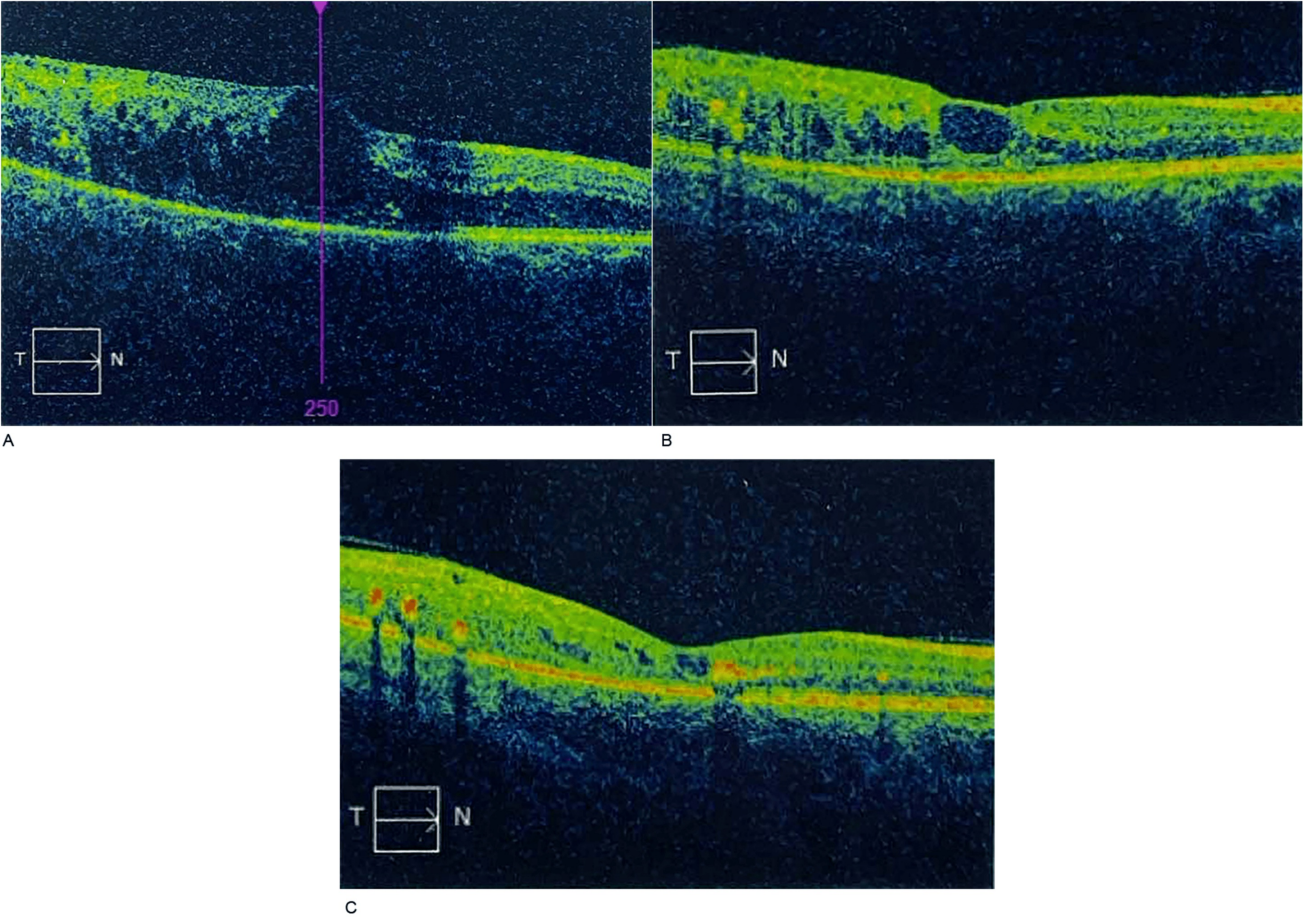
Visual representation (OCT scans: T→N) of treatment response in Patient 4. (A) Baseline: central macular thickness of 487 µm; (B) Follow-up at 1 month: thickness of 350 µm; (C) Follow-up at 2 months: thickness of 282 µm. T→N: Temporal to nasal orientation; OCT: Optical Coherence Tomography.

This patient had a complex cardiometabolic profile (25-year DM, post-percutaneous transluminal coronary angioplasty for coronary artery disease (CAD)) with severe baseline parameters (BCVA 1/60, CMT 487 μm). She presented with a 2-month history of diminution of vision and was diagnosed with DME. At baseline, BCVA was 1/60, and CMT was 487 μm. Faricimab therapy achieved progressive recovery: BCVA 6/60 (1 month) with CMT decreasing to 350 μm, BCVA 6/24 (2 months) with CMT decreasing to 282 μm, and BCVA 6/12 (3 months) with CMT improving to 209 μm. The patient was on concomitant oral hypoglycemics, antihypertensives, aspirin, Nevanac, and moxifloxacin eye drops. Initially, she reported little satisfaction (score 3), which improved to moderately satisfied (score 2) at 1- and 2-month follow-ups, and further to very satisfied (score 1) by the end of the study. No AEs were reported. The case is visually represented in Figure 4.

Case 5

**Figure 5:**
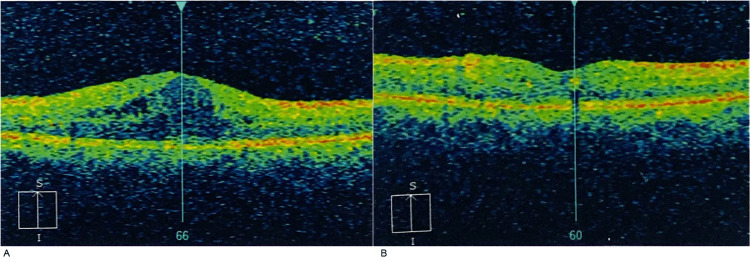
Visual representation (OCT scans: T→N) of treatment response in Patient 5. (A) Baseline: central macular thickness of 565 µm; (B) Follow-up at 1 month: thickness of 339 µm. S→I: Superior to inferior; OCT: Optical Coherence Tomography.

This patient presented with a 1-month history of diminished vision. He had DM for 1 year and was diagnosed with DME. At baseline, BCVA was 6/18, and CMT was 565 μm. He received monthly faricimab injections. At 1 month, BCVA improved to 6/12, with CMT decreasing to 339 μm. By 2 months, BCVA further improved to 6/9, and CMT reduced to 312 μm. At 3 months, BCVA reached 6/6, with CMT dropping to 279 μm. The patient was on concomitant oral hypoglycemics, Nevanac, and moxifloxacin eye drops. He initially reported being moderately satisfied (score 2). After the first dose, his satisfaction improved to very satisfied (score 1) and remained consistent through all follow-up points. No AEs were reported. The case is visually represented in Figure 5.

## Discussion

**Figure 6:**
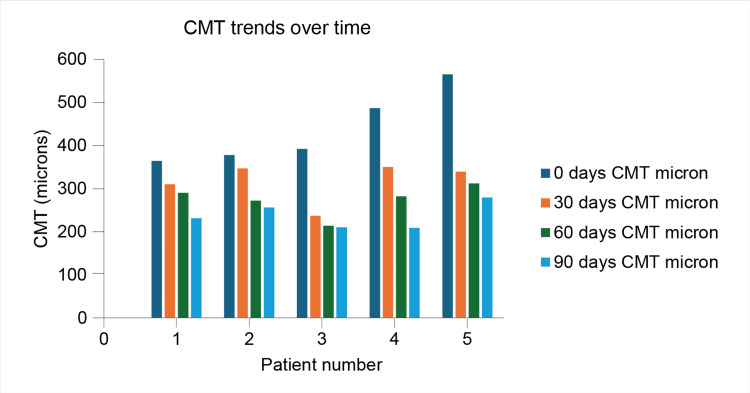
CMT trends over time. CMT: Central macular thickness.

This case series explored the efficacy and safety of a monthly loading dose regimen of faricimab in treatment‐naïve Indian patients with DME. We found that faricimab led to notable anatomical improvements, reflected by reductions in CMT (Figure 6), and corresponding functional gains in BCVA over 3 months. Notably, the AEs recorded were minimal, and no ocular AEs, such as intraocular inflammation or increased intraocular pressure, were observed throughout the study in any of the cases. The present case series highlights how faricimab aids in controlling disease progression in DME and in reducing the treatment burden typically associated with frequent intravitreal injections.

Currently, treating DME with anti-VEGF agents requires frequent appointments with healthcare providers, which can lead to undertreatment. Consequently, real-world clinical outcomes often fall short of the clinical responses seen in trials [11]. Faricimab simultaneously inhibits both VEGF-A and Ang-2, reducing vascular permeability and enhancing vascular stability, leading to rapid and sustained improvements in retinal morphology [6,8]. Moreover, the potential to extend treatment intervals could alleviate the injection burden commonly seen with conventional anti-VEGF therapies. This aspect is particularly important in clinical settings like India, where resource constraints and patient compliance are significant considerations.

In the reported cases, the improvements observed were consistent with results from pivotal clinical trials and recent real-world studies. For instance, the YOSEMITE and RHINE trials reported non-inferior vision gains with faricimab relative to aflibercept, along with superior anatomical outcomes and the possibility of extended dosing intervals [7,12]. The one-year results from the YOSEMITE and RHINE trials (both phase III randomized trials) reported non-inferiority of faricimab, as assessed using ETDRS letters every 8 weeks, compared with aflibercept [13]. Faricimab improved vision and retinal anatomy in patients with DME when administered with adjustable dosing intervals of up to 16 weeks, suggesting that faricimab may offer a more durable treatment option [13]. Wong TY et al. highlighted the two-year results from YOSEMITE and RHINE. While efficacy remained similar to the one-year results, faricimab showed improved durability in the second year of these trials. In the T&E groups, more patients were able to extend their dosing intervals while still achieving similar visual acuity improvements and better anatomical outcomes compared to those receiving aflibercept [11]. The findings indicate that Ang-Tie signaling plays a role in vascular stability, supporting the inference that dual inhibition of Ang-2 and VEGF-A, compared to VEGF inhibition alone, may enhance treatment durability [11]. Additionally, the median number of faricimab T&E injections over two years was lower than in previous studies using pro re nata dosing, suggesting that this dosing strategy could reduce the frequency of visits and injections for patients [11].

The TAHOE trial noted improvements in visual acuity (as measured in ETDRS letters) and central subfield thickness parameters for both treatment-naïve and previously treated patients with DME who received at least one intravitreal injection in a real-world setting [12]. FARETINA-DME, a retrospective real-world study, analyzed 3,961 eyes from 2,692 patients treated with faricimab for DME. On average, patients received 2.6 ± 1.3 faricimab injections over a follow-up period of 55.1 ± 47.2 days. Vision remained stable in previously treated eyes, while treatment-naïve eyes showed a mean improvement of 3 letters after four initial injections. Notably, 61.8% of previously treated patients achieved a treatment interval extension of 6 weeks or more after just 1-2 injections [12]. Additionally, the FARWIDE-DME study reported that treatment-naïve eyes achieved a mean extension to an 8-week interval after the fourth faricimab injection, with roughly 64% of naïve eyes and 44% of previously treated eyes maintaining intervals of 8 weeks or more [12].

Real-world data suggest that many patients with DME do not adhere to the recommended dosing schedule, potentially resulting in suboptimal visual outcomes. This underscores the need for therapies that improve vision while minimizing treatment burden by offering extended durability [14]. A network meta-analysis highlighted that faricimab in a T&E regimen significantly reduced retinal thickness and improved BCVA compared to other flexible dosing strategies. Compared with flexible dosing regimens, the faricimab T&E regimen resulted in a statistically significant reduction in retinal thickness, achieving superior retinal drying (55-125 µm) [9]. Moreover, a lower injection frequency was noted in the T&E regimen than in other flexible dosing regimens, with a reduction of 0.92-1.43 injections [9].

The present case series exclusively analyzed an Indian cohort, which has been reported only sparsely. A retrospective analysis at four centers in India involving 39 eyes (16 treatment-naïve and 23 recalcitrant) treated with intravitreal faricimab found that both groups experienced significant improvements in BCVA, with larger gains in the treatment-naïve group [10]. A significant reduction in CMT at 6 months was also noted, with the naïve group achieving a more pronounced decrease [10].

Our report indicates that faricimab demonstrated a favorable safety profile, with no significant AEs observed during the 3-month follow-up period. This aligns with previous studies indicating low ocular complication and intraocular inflammation rates with faricimab therapy. One-year results from the YOSEMITE and RHINE trials reported a comparable incidence of ocular AEs between faricimab and aflibercept administered every 8 weeks [13]. Furthermore, the 2-year results from the YOSEMITE and RHINE trials showed that the incidence of ocular AEs, most of which were mild or moderate, was similar between patients receiving faricimab (in both the 8-week and T&E arms) and those receiving aflibercept [11]. The rates of serious ocular AEs and non-ocular AEs were also comparable between the two treatment groups. Additionally, intraocular inflammation events (vitreitis, uveitis, chorioretinitis, keratic precipitates) were low and similar among patients. These events were deemed unrelated to the study drugs, faricimab and aflibercept [11]. The TAHOE trial also reported zero cases of intraocular inflammation, vasculitis, endophthalmitis, or retinal artery occlusion in patients treated with faricimab in a real-world setting [12].

A closer examination of individual cases in our report suggests that patients with a shorter duration of diabetes experienced better responses to faricimab. Furthermore, patients with better baseline visual acuity tended to respond more rapidly to treatment. For instance, Patient 5, despite having a baseline BCVA of 6/18 and a very high CMT (565 μm), achieved complete visual recovery (6/6) and a reduction in CMT to 279 μm. In contrast, patients with long-standing diabetes and additional comorbidities, such as HTN or CAD, showed more gradual improvement, though still a clinically notable response. For example, Patient 2 (a 58-year-old female) with a 13-year history of diabetes, HTN, and CAD, improved from 6/60 to 6/6 BCVA and showed a reduction in CMT from 378 μm to 256 μm despite her chronic systemic conditions. Patient 4 (a 54-year-old female), despite a poor baseline BCVA (1/60) and CMT (487 μm) and a complex cardiometabolic profile, improved significantly to 6/12 BCVA and achieved a CMT of 209 μm. Patients with baseline CMT values above 400 μm (Patients 4 and 5) exhibited notable reductions in macular thickness, reaching near-normal values by the final visit. These differences underscore the importance of early intervention in DME management and suggest that patient-specific factors may influence treatment outcomes.

This case series has certain limitations, including a small sample size, its retrospective design, and a short follow-up period. Therefore, the results should be interpreted with caution. Larger-scale, randomized controlled trials are needed to confirm the efficacy and safety of faricimab across diverse populations, assess its long-term durability, and determine the optimal dosing strategy. Comparative studies evaluating faricimab against other anti-VEGF agents in both treatment-naïve and recalcitrant DME cases would also help refine treatment algorithms and identify patient subgroups most likely to benefit from the therapeutic approach explored in the present study.

## Conclusions

This case series supports the use of faricimab as a treatment option for patients with treatment-naïve DME. It demonstrates significant anatomical and functional benefits, along with a well-tolerated safety profile. Dual inhibition of VEGF-A and Ang-2 led to notable improvements, with faster anatomical responses observed in patients with a shorter duration of diabetes. These encouraging findings highlight the potential of faricimab, and further research involving larger cohorts and longer follow-up periods will help refine its optimal use in routine clinical practice.
